# Development of a machine learning framework for radiation biomarker discovery and absorbed dose prediction

**DOI:** 10.3389/fonc.2023.1156009

**Published:** 2023-05-15

**Authors:** Björn Andersson, Britta Langen, Peidi Liu, Marcela Dávila López

**Affiliations:** ^1^ Bioinformatics Core Facility, The Sahlgrenska Academy, University of Gothenburg, Gothenburg, Sweden; ^2^ Department of Radiation Oncology, Division of Molecular Radiation Biology, University of Texas (UT) Southwestern Medical Center, Dallas, TX, United States

**Keywords:** ionizing radiation, radionuclides, absorbed dose, biomarkers, transcriptomics, kNN (k nearest neighbor), caret

## Abstract

**Background:**

Molecular radiation biomarkers are an emerging tool in radiation research with applications for cancer radiotherapy, radiation risk assessment, and even human space travel. However, biomarker screening in genome-wide expression datasets using conventional tools is time-consuming and underlies analyst (human) bias. Machine Learning (ML) methods can improve the sensitivity and specificity of biomarker identification, increase analytical speed, and avoid multicollinearity and human bias.

**Aim:**

To develop a resource-efficient ML framework for radiation biomarker discovery using gene expression data from irradiated normal tissues. Further, to identify biomarker panels predicting radiation dose with tissue specificity.

**Methods:**

A strategic search in the Gene Expression Omnibus database identified a transcriptomic dataset (GSE44762) for normal tissues radiation responses (murine kidney cortex and medulla) suited for biomarker discovery using an ML approach. The dataset was pre-processed in R and separated into train and test data subsets. High computational cost of Genetic Algorithm/k-Nearest Neighbor (GA/KNN) mandated optimization and 13 ML models were tested using the caret package in R. Biomarker performance was evaluated and visualized *via* Principal Component Analysis (PCA) and dose regression. The novelty of ML-identified biomarker panels was evaluated by literature search.

**Results:**

Caret-based feature selection and ML methods vastly improved processing time over the GA approach. The KNN method yielded overall best performance values on train and test data and was implemented into the framework. The top-ranking genes were *Cdkn1a*, *Gria3*, *Mdm2* and *Plk2* in cortex, and *Brf2*, *Ccng1*, *Cdkn1a*, *Ddit4l*, and *Gria3* in medulla. These candidates successfully categorized dose groups and tissues in PCA. Regression analysis showed that correlation between predicted and true dose was high with R^2^ of 0.97 and 0.99 for cortex and medulla, respectively.

**Conclusion:**

The caret framework is a powerful tool for radiation biomarker discovery optimizing performance with resource-efficiency for broad implementation in the field. The KNN-based approach identified *Brf2*, *Ddit4l*, and *Gria3* mRNA as novel candidates that have been uncharacterized as radiation biomarkers to date. The biomarker panel showed good performance in dose and tissue separation and dose regression. Further training with larger cohorts is warranted to improve accuracy, especially for lower doses.

## Introduction

Ionizing radiation is an essential tool in the vast majority of cancer treatments, either therapeutically, diagnostically or adjuvant. Accurate knowledge of the relationship between a certain dose of ionizing radiation and biological effect is paramount for successful treatment planning and risk assessment. Radiation biomarkers are an emerging tool that reflect dose-response or predict acute or long-term outcomes after therapeutic, accidental, or occupational exposures (1–4). While high-throughput techniques such as RNA sequencing and mass spectrometry-based protein quantitation have been increasingly used in the emerging discipline, biomarker identification in large-scale gene expression data has been limited by lack of analytical tools that adequately process the complexity of so-called omics data. Machine Learning (ML) approaches can overcome this limitation and vastly improve the robustness and throughput in biomarker screening. In particular, ML approaches prioritizes predictive power of biomarker candidates (genes, protein, transcripts, or probes etc.) over functional association or differential expression (fold-change). Moreover, a bottom-up approach can detect relevant gene regulation in multi-dimensional space without being limited by pre-determined canonical pathways or functional association for a given stressor.

In the field of radiation research, a bottom-up approach is particularly advantageous since key phenomena of biological radiation effects are still topics of academic discourse where the field has struggled to find clear consensus. A prominent example are dose thresholds, meaning for instance whether certain low dose exposures can be considered inconsequential or safe, as discussed in detail by (5–10). A bottom-up approach to genome-wide expression data analysis can unravel the complexity of biological responses and identify molecular markers based on statistical features alone without the analysis being limited by, e.g., pre-supposed (canonical) pathways, (manual) database curation for enrichment analysis, or presumed radiation dose categories.

Currently, mostly conventional analytical approaches have been used for radiation biomarker discovery building on differentially expressed gene (DEG) profiles, which poses critical limitations on throughput, sensitivity, and specificity (11). ML has emerged in radiation oncology and medicals physics as a technology to overcome these limitations, but its application to radiation biomarker screening and basic radiation biology is underexplored (12–19). Early implementation of ML approaches into radiation biomarker discovery workflows can be of tremendous benefit to the field: ML-based frameworks can vastly reduce the analytical time burden for researchers, increase analytical certainty (accuracy) through improved specificity and sensitivity, and help establish consensus guidelines for sample and data acquisition, such as group size, replicates, batch effects, etc.

To the best of our knowledge, this work is the first implementation of ML approaches for radiation biomarker discovery for normal tissue exposure. The aim was to develop a resource-efficient ML framework for detection of high-sensitivity and high-specificity biomarker candidates within genome-wide expression (omics) datasets. We performed a strategic literature search to identify a suitable radiobiology dataset and implemented the Genetic Algorithm combined with the k-Nearest Neighbors (GA/KNN) approach as proof-of-concept. For framework optimization, we tested thirteen different ML methods using the caret package in R to determine optimal performance on such datasets and implement into the framework. The top-ranking radiation biomarker candidates (mRNA probes) were also evaluated in their radiobiological context by strategic literature search.

## Methods

### Strategic literature searches

**Figure 1:**
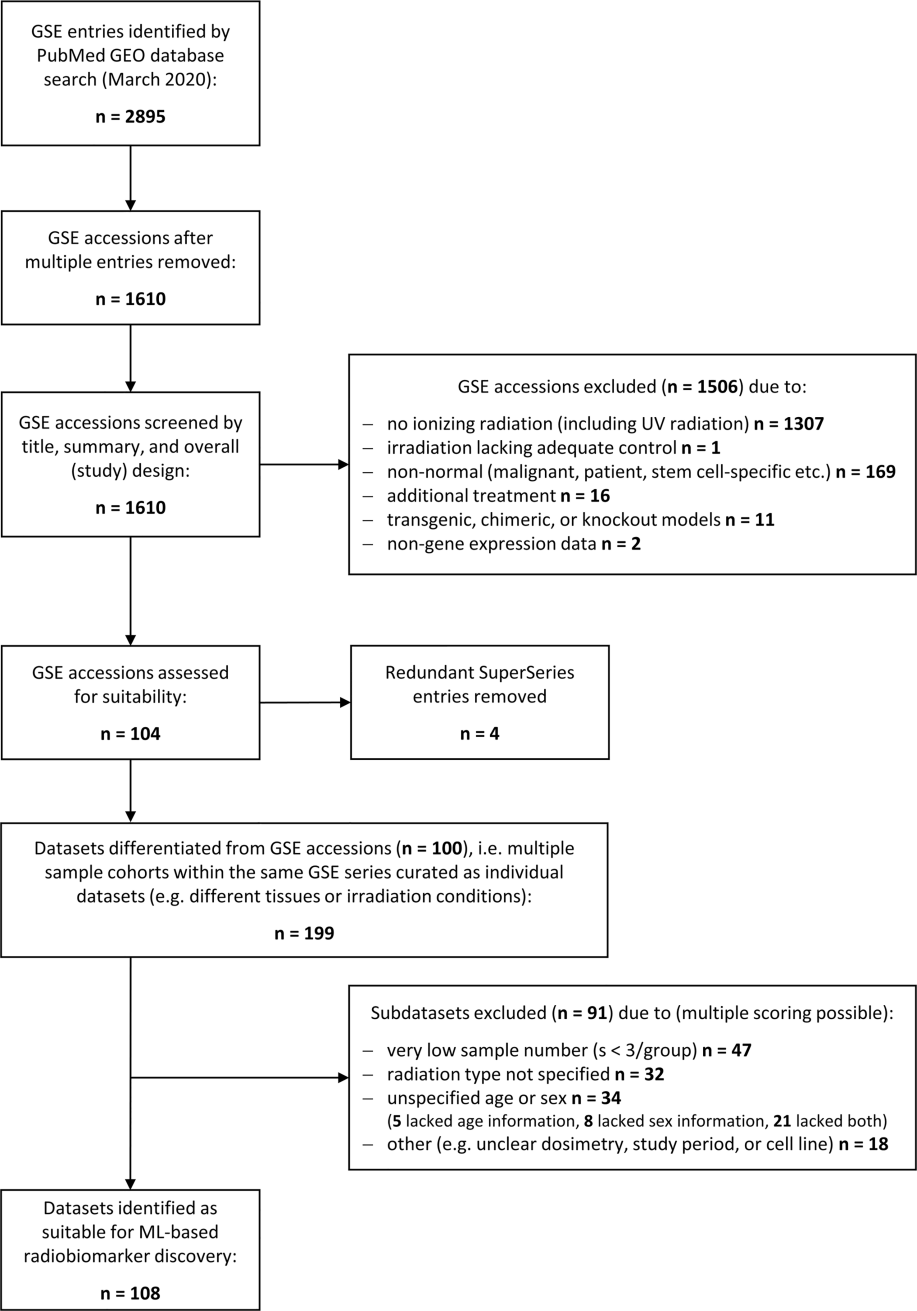
Schematic illustrating the database search to identify gene expression datasets for normal tissue radiation responses. Flowchart showing the database search performed at the National Center for Biotechnology Information (NCBI) Gene Expression Omnibus (GEO) resource (https://www.ncbi.nlm.nih.gov/geo/). The search was performed in March 2020 yielding 2895 transcriptional gene expression entries (mRNA, miRNA, lncRNA etc.) that consisted to a large part of multiple entries. The remaining 1610 unique accessions were screened to identify normal tissue datasets from any mammalian species subjected to any type of ionizing radiation (X-rays, alpha or beta particles, heavy ions, etc.), any source (external beams, (partially) sealed radioactive sources, or injected radionuclides), and any form of exposure (external, internal, whole body or partial body irradiation, etc.). Most of the accession numbers were unsuitable for normal tissue biomarker identification and excluded. The remaining accessions were manually curated to delineate subsets of data with insufficient data quality such as low sample number, unspecified dosimetry, or time points. In total, 108 suitable datasets for ML processing were identified.

A literature search was performed using the National Library of Medicine at https://www.ncbi.nlm.nih.gov/gds/ to identify suitable gene expression datasets. Flow diagram showing the database search and selection procedure (**Figure 1**).

The search string (“gene expression signature”[All Fields] OR “gene expression”[All Fields] OR “gene expression changes”[All Fields] OR “transcription”[All Fields] OR “biomarker”[All Fields]) AND (“radiation exposure”[All Fields] OR “ionizing radiation” [All Fields] OR “radiotherapy”[All fields] OR “irradiation”[All fields]) AND (“microarray”[All Fields] OR “RNA-seq”[All Fields] OR “sequencing”[All Fields]) AND (“Mus musculus”[Organism] OR “Rattus norvegicus”[Organism] OR “Homo sapiens”[Organism]) was used to identify relevant transcriptomic datasets from animal and human cohorts exposed to any type and modality of ionizing radiation and any tissue or cell type. The search was performed in the repository Gene Expression Omnibus (GEO) at https://www.ncbi.nlm.nih.gov/gds on January 26, 2023.

To evaluate the novelty of ML-based biomarkers candidates, the database was searched at https://pubmed.ncbi.nlm.nih.gov/ (PubMed) with a broad and narrow search string. Search string 1 "(gene symbol) AND ((ionizing) OR (radiation)) AND ((biomarker) OR (absorbed dose) OR (dose) OR (dose response))" was used to determine whether a gene was previously proposed specifically for use in biodosimetry. Search string 2 "(Plk2) AND ((ionizing) OR (radiation))" was used to determine whether a gene had been previously associated with any form of radiation research in the literature base. Both search strings were used on January 26, 2023. In addition, the candidates were cross-referenced with the radiation biomarker lists composed by Snyder & Morgan (20) and Chaudhry (21) and conventional DEG analysis reported by Schüler et al. (22).

### Data cohort

The transcriptomic microarray dataset GSE44762 was chosen as a suitable dataset from the GEO series accession (GSE) database search; the complete study design and results from conventional (DEG) analysis were first described elsewhere (22). In brief, female BALB/c nude mice (n = 3/group) were intravenously injected with 1.3, 3.6, 14, 45, and 140 MBq ^177^Lu-octreotate in physiological saline, which resulted in absorbed dose to the kidneys (tissue of interest) of 0.13, 0.34, 1.3, 4.3, and 13 Gy over 24 hours. The control group received physiological saline only (i.e. 0 Gy). At 24 hours, the animals were euthanized and the kidneys were excised and flash-frozen in liquid nitrogen. Kidney cortex and kidney medulla tissues were dissected in dry ice and mRNA was extracted, quality-assured, and subjected to microarray analysis on MouseRef-8 Whole-Genome Expression Beadchips (Illumina). Samples were analyzed individually (non-pooled) and the total cohort consisted of 36 samples. Input variables for ML methods are Illumina probe identifiers representing microarray probe sequences targeting mRNA molecules (or fragments thereof) for detection and semi-quantitative analysis.

### Statistical analysis and machine learning methods

All the analyses were performed using RStudio Server with R version 4.0.2 (http://www.r-project.org) (23). For basic statistical analysis, base R and tidyverse functions were used. For the machine learning calculations, all models were trained and evaluated using the caret (Classification and Regression Training) package version 6.0-86 (24).

The following ML methods were evaluated: C5.0 Decision Tree, CART, Lasso/Elastic Net, KNN, GBM, Logistic Regression, Naive Bayes, NNET, PLS, Penalized Discriminant Analysis, Random Forest, SIMCA, and SVM. For Principle Component Analyses (PCA), the data was scaled and centered; PCA was used to identify potential confounders and/or deviating samples and assess performance. To evaluate the dose response, cross validated multi variable regression models were created after log-transformation of the radiation biomarkers.

### Data pre-processing

Prior to model creation, the data were inspected and pre-processed for analysis. For data pre-processing the R-package tidyverse was used in general. Rows with not significant detection p-value, all NAs and zero variance predictors were removed. Quantile normalization (R-package preprocess Core), scaling and centering were performed on the variables. To remove redundant and irrelevant features, as well as speed up the algorithm testing an optimization, the most important genes were selected by first running feature selection with the Recursive Feature Elimination (RFE) rfe-function from the caret package. RFE makes it possible to identify an ML model with less features but with similar model performance. Feature selection was used to reduce dimensionality, minimize noise and prevent collinearity. Recursive Feature Elimination (RFE), was performed using the rfe-function from the caret package, Cohen’s kappa was used for the selection of the optimal subset based on size and performance with 10-fold cross-validation, repeated 5 times using the random forest variable importance selection. If two sets of features had similar kappa the smaller one was selected.

### Model testing and evaluation

To construct a model, the pre-processed data were split into a train and a test set by randomly allocating 70% of cases to the training data subset and 30% for the testing data subset. For proof-of-concept, the Genetic Algorithm (GA) and k-Nearest Neighbor (GA/KNN) approach was implemented as described elsewhere (18). For optimization, all ML models listed above were tested and evaluated using the caret package in R. To ensure stable and well performing models Repeated k-fold cross-validation (CV) was used with k = 5 and 20 repeats. Due to imbalanced datasets, Cohen’s kappa was selected as the main metric for evaluating the performance of the classification models. Sensitivity and specificity were considered by creating confusion matrixes (package e1071) for both the train and test set and analyzed based on the corresponding predictions and variable importance (VIP) rankings were calculated on applicable models.

### Statistical analysis and visualization

Univariate analysis revealed the statistical significance p < 0.05 of the top-ranking variables (transcripts). These were used as input for the PCA plots as described elsewhere (18). Multivariable dose regression was performed on both kidney cortex and medulla tissue subsets, in similar manner as for classification above using log-transformed radiation dose values. The caret rfe-function was used for feature selection (10-fold cross-validation, repeated 5 times), followed by using 10-fold cross-validated linear regression on the train set and evaluated using the test set.

### Biomarker candidate evaluation

Top-ranked ML-based biomarker candidates were used in PCA to visualize classification performance by dose group and tissue. The candidates were also used in dose regression to determine performance of dose prediction with tissue-specificity. To evaluate the novelty of ML-based biomarker candidates, strategic literature searches were performed on all KNN-based candidates with additional searches in biomarker panel reviews and previous DEG analysis (20–22). A novelty score from 0 to 2 was applied with regard to literature documentation: 0 for genes discussed as biomarker candidates in vast literature; 1 for genes proposed as candidates in some literature; 2 for genes not yet proposed as candidates in literature.

## Results

We performed a strategic literature search to frame the field of omics-based radiation biomarker discover and to identify suitable RNA-based expression datasets allowing for feature combination. Around 1,600 unique transcriptional gene expression datasets with mRNA, micro-RNA, long non-coding RNA, or total RNA have been deposited at the Gene Expression Omnibus in conjunction with the term ‘radiation’ (**Figure 1**). However, more than 1,300 entries did not use ionizing radiation and over 160 entries comprised some form of inadequate normal tissue model such as non-healthy patient-derived or stem-cell specific. After removing all irrelevant GSE accessions and redundant super series, we obtained 104 suitable accessions that were manually curated to create data subsets whenever multiple features were used in the same accession, such as different radiation sources or exposure conditions, male or female specimen or different age groups, or different tissues analyzed within the same cohort. Upon curation, around 199 subsets were composed for normal tissue ionizing radiation biomarker screening. These subsets were further assessed for quality and 91 subsets were excluded with insufficient information, e.g., unspecified radiation type, age, or sex, or sample number less than 3. Among the remaining 108 data subsets, we chose GSE44762 from murine kidneys since it offered two tissues (cortex and medulla) obtained from the same organ and specimen.

### ML approaches

**Figure 2:**
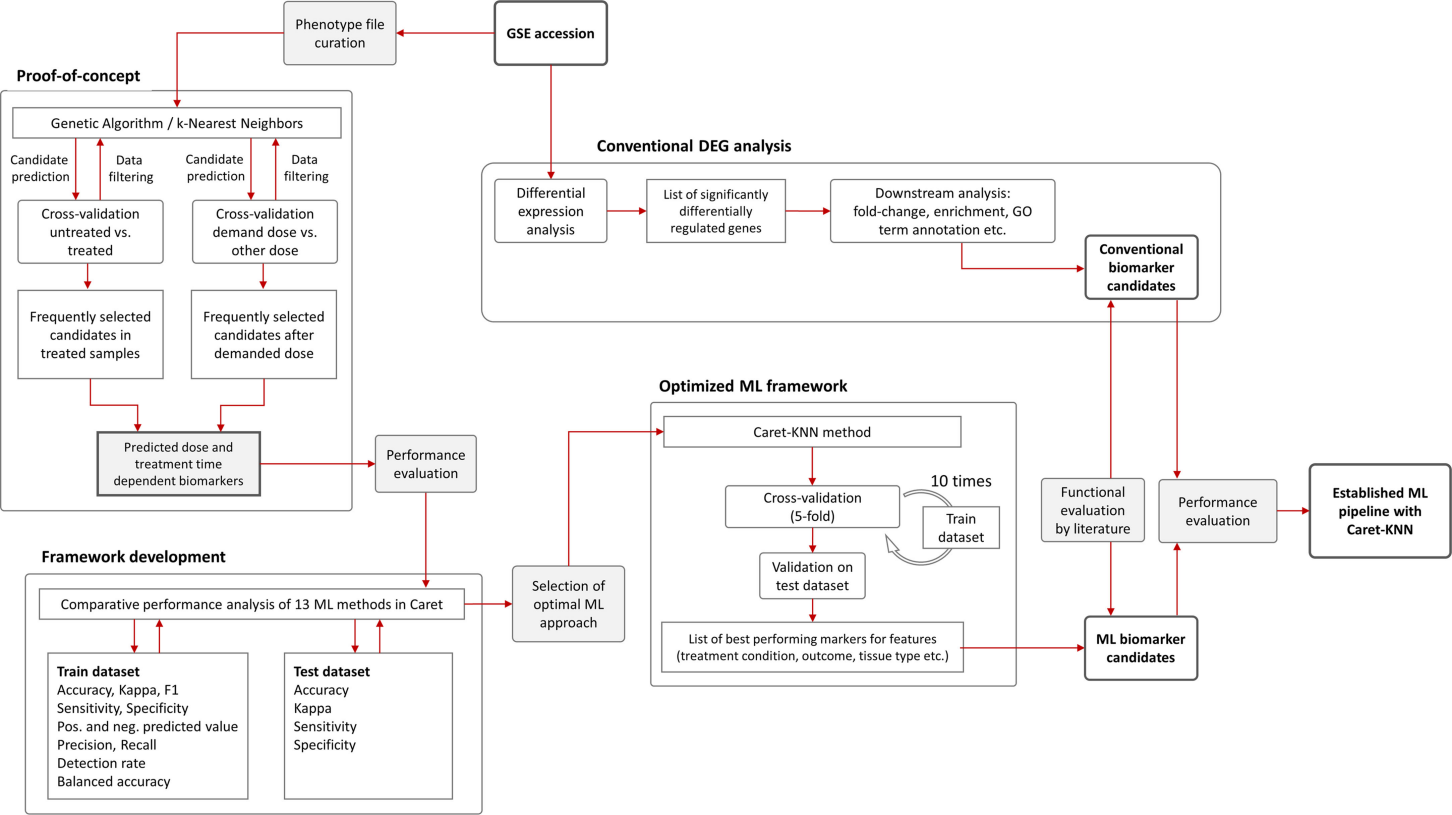
Flowchart of machine learning approaches and gene expression analysis. The Genetic Algorithm/k-Nearest Neighbors (GA/KNN) approach as described elsewhere (18) was used to implement the proof-of-concept using the GSE44762 transcriptomic microarray dataset. The previous DEG analysis is described elsewhere (22). To develop a framework and decrease resource demand, the caret package in R was used and thirteen ML algorithms were performance-tested on training (0.7 ratio) and test (0.3 ratio) data subsets created from GSE44762, i.e. Logistic Regression, Genetic Algorithm/k-Nearest Neighbors, Penalized Discriminant Analysis, Elastic Net, C5.0 Decision Tree, Soft Independent Modelling by Class Analogy, Partial Least Squares, Naive Bayes, Random Forest, Neural Networks, Gradient Boosting Machine, Classification And Regression Trees (CART), and Support Vector Machine. The KNN method outperformed all other ML methods and optimized the ML framework by drastically reducing computational cost compared with original GA/KNN implementation and was integrated into the ML framework as illustrated.

After pre-processing of the data, 36 samples and 9783 variables remained. (Please note the initial dataset contained 25,697 features (genes) that were filtered for i) delete rows that have detection p-value > 0.05 and ii) columns that have all NAs (also samples that have only 0 as expression values). These were used as input with a randomly assigned 0.7 train to 0.3 test ratio for the respective models; for analysis between tissues we used one tissue as train and one for test. The assignment of the data into train and test datasets was stratified using the function create Data Partition from the R package caret. The project workflow from phenotype file creation over proof-of-concept to pipeline optimization and final implementation is illustrated in **Figure 2**. GA/KNN as proof-of-concept was first implemented following Li and co-authors (18) and successfully identified 9 biomarker candidates (12 probes) for kidney cortex and 3 candidates (3 probes) for kidney medulla (**Table 1**). Among these candidates, only Per2 was previously identified by conventional DEG analysis as reported elsewhere (22); rendering the vast majority of KNN-based biomarkers as novel hits that were not identified by conventional DEG analysis.

**Table 1:** Radiation biomarker candidates in murine kidneys identified *via* DEG analysis or GA/KNN approach.

	Identified in Tissue		Identified via	
Gene Symbol	Kidney Cortex	Kidney Medulla	Conventional DEG Analysis*	GA/KNN Approach
*Actb*		x	x	
*Adipoq*		x	x	
*Angptl4*	x	x	x	
*BC052040*	x			ILMN_1227874
*Car3*	x	x	x	
*Ccng1*	x			ILMN_2500276
				ILMN_2702233
*Cdkn1a*	x	x	x	
*Cfd*		x	x	
*Ckb*		x	x	
*Cyp24a1*	x	x	x	
*Cyp27a1*	x	x		Cortex: ILMN_2620326, ILMN_2960108
				Medulla: ILMN_2960114
*Dbp*	x	x	x	
*Gdf15*	x	x	x	
*Havcr1*	x	x	x	
*Hes1*		x		ILMN_1247691
*Hist1h4j*	x			ILMN_1256989
*Hmgcs2*	x	x	x	
*Mup2*	x		x	
*Nfil3*	x			ILMN_2595732
*Nupr1*		x	x	
*Per2*	x	x	x	Cortex: ILMN_298786, ILMN_2987862
*Rbm4*	x			ILMN_2769133
*Rtkn*	x			ILMN_3143266
*S100a6*	x		x	
*Slc12a6*	x			ILMN_2716511
*Tef*		x		x
*Upk1b*	x	x	x	Medulla: ILMN_2676052

List of genes identified in transcriptomic microarray data (GSE44762) from murine kidney cortex and medulla tissues irradiated with 0.13–13 Gy absorbed dose from i.v. injected ^177^Lu-octreotate over 24 hours including unirradiated controls.

DEG, differentially expressed gene(s); x, identified; void, not identified; ILMN, Illumina probe identifier.

*as previously reported in Schüler et al., 2014 (22); please note ILMN identifiers were not given in the publication.

Despite candidate performance, a key disadvantage of the original Genetic Algorithm-based KNN implementation was its high resource demand in terms of processor time. The GA/KNN method is a C program language implementation and computationally intensive, as it searches for many near-optimal solutions (chromosomes) based on the nature of randomness. For example, a typical run would take several weeks on a 16 GB RAM Intel Xenon server even for a small number of samples. To resolve this bottleneck and lower resource barriers for broad implementation in the field, we optimized the ML pipeline and tested several different ML models using the resource-friendly caret package in R and compared their performance using a set of parameters such as accuracy, kappa, sensitivity, specificity, prediction values, and detection rate (Please see **Table 2** for more details and performance of all models). In contrast to GA/KNN, the RFE/caret framework could be run in just a few seconds after the RFE step. Thirteen models were built including the caret-based KNN algorithm, i.e. C5.0 Decision Tree, Classification And Regression Trees (CART), Lasso/Elastic Net, Gradient Boosting Machine (GBM), Logistic Regression, Naive Bayes, Neural Networks (NNET), Partial Least Squares (PLS), Penalized Discriminant Analysis, Random Forest, Soft Independent Modelling by Class Analogy (SIMCA), and Support Vector Machine (SVM). The models were built with all the variables, irrespective of their statistical significance. When analyzing the variable importance of the two best performing models, the results were generally similar to those of the univariate analysis (data not shown). Optimal biomarker panel size was determined by resampling performance over subset size. For dose prediction, acceptable Root Mean Square Error (RMSE) values below 30.0 were achieved by panels between 3 and 25 variables. The lowest RMSE was achieved by the 10-variable panel (23.83) closely followed by the 5-variable panel (24.20) (**Table 3**). Similarly, for tissue prediction, RMSE was lowest for the 10-variable panel (0.9733) followed by the 5-variable panel (0.9700) (**Table 4**).

**Table 2:** Performance results of different machine learning algorithms.

	Logistic Regression	k-Nearest Neighbors (KNN)	Penalized Discriminant Analysis (PDA)	Lasso/ Elastic Net	C5.0 Decision Tree	Soft Independent Modelling by Class Analogy (SIMCA)	Partial Least Squares (PLS)	Naive Bayes	Random Forest	Neural Networks (NNET)	Gradient Boosting Machine (GBM)	Classification and Regression Trees (CART)	Support Vector Machine (SVM)
Measure	m_glm	m_kknn	m_pda	m_glmnet	m_C5	m_CSimca	m_pls	m_nb	m_parRF	m_nnet	m_gbm	m_rpart	m_svm
Train data													
Accuracy	0.56	0.86	0.87	0.87	0.74	0.42	0.87	0.87	0.80	0.78	0.78	0.75	0.79
Kappa	0.11	0.47	0.54	0.53	0.24	0.10	0.54	0.48	0.00	0.40	0.40	0.20	0.00
F1	0.43	0.67	0.73	0.68	0.52	0.40	0.70	0.70	N/A	0.59	0.59	0.49	N/A
Sensitivity	0.62	0.43	0.56	0.49	0.42	0.85	0.53	0.42	0.00	0.62	0.62	0.36	0.00
Specificity	0.54	0.97	0.96	0.98	0.83	0.31	0.97	0.99	1.00	0.82	0.82	0.86	1.00
Pos_Pred_Value	0.27	0.85	0.82	0.90	0.42	0.28	0.86	0.98	N/A	0.53	0.53	0.40	N/A
Neg_Pred_Value	0.86	0.87	0.89	0.88	0.84	0.93	0.89	0.87	0.80	0.89	0.89	0.84	0.79
Precision	0.27	0.85	0.82	0.90	0.42	0.28	0.86	0.98	N/A	0.53	0.53	0.40	N/A
Recall	0.62	0.43	0.56	0.49	0.42	0.85	0.53	0.42	0.00	0.62	0.62	0.36	0.00
Detection_Rate	0.13	0.09	0.12	0.10	0.09	0.18	0.11	0.09	0.00	0.13	0.13	0.08	0.00
Balanced_Accuracy	0.58	0.70	0.76	0.73	0.63	0.58	0.75	0.71	0.50	0.72	0.72	0.61	0.50
Test data													
Accuracy	0.48	0.86	0.81	0.86	0.67	0.52	0.86	0.86	0.86	0.81	0.81	0.81	0.81
Kappa	-0.02	0.49	0.00	0.35	0.01	0.21	0.35	0.35	0.35	0.00	0.00	0.24	0.00
Sensitivity	0.50	0.50	0.00	0.25	0.25	1.00	0.25	0.25	0.25	0.00	0.00	0.25	0.00
Specificity	0.47	0.94	1.00	1.00	0.76	0.41	1.00	1.00	1.00	1.00	1.00	0.94	1.00

Thirteen different ML approaches were tested using train and test data subsets created from transcriptomic microarray (GSE44762) data from kidney cortex and medulla irradiated with 0.13 – 13 Gy absorbed dose from i.v. injected ^177^Lu-octreotate over 24 hours including unirradiated controls.

N/A, not available.

**Table 3:** Resampling performance over subset size for recursive feature selection for dose biomarkers.

Variables	RMSE	MAE	RMSE SD	MAE SD
1	32.65	28.83	30.36	27.05
2	30.80	26.50	27.75	24.00
3	27.41	23.64	25.15	20.73
4	24.84	21.59	22.96	19.49
5	24.20	20.83	22.74	19.34
10	23.83	20.67	19.39	16.99
15	25.47	21.92	18.72	15.81
20	27.29	24.31	19.38	17.09
25	27.81	24.76	20.14	17.49
9783	40.83	38.02	23.07	21.06

The accuracy of ML model performance in dependence of variable panel size is shown for cortex. Results for medulla were similar (not shown). Variables refer to microarray probes.

MAE, Mean Absolute Error; RMSE, Root Mean Square Error; SD, standard deviation.

**Table 4:** Resampling performance over subset size for recursive feature selection for tissue biomarkers.

Variables	Accuracy	Kappa	Accuracy SD	Kappa SD
1	0.9033	0.800	0.19660	0.4041
2	0.9333	0.868	0.16836	0.3347
3	0.9600	0.920	0.13702	0.2740
4	0.9700	0.940	0.11995	0.2399
5	0.9700	0.940	0.11995	0.2399
10	0.9733	0.940	0.10838	0.2399
15	0.9633	0.920	0.12729	0.2740
20	0.9633	0.920	0.12729	0.2740
25	0.9633	0.920	0.12729	0.2740

The accuracy of ML model performance in dependence of variable panel size is shown. Variables refer to microarray probes.

Kappa, Cohen’s kappa; SD, standard deviation.

A 5-variable panel was chosen as the optimal panel size since a factor of 2 results in a large reduction of experimental cost for validation or clinical implementation at comparable RMSE. (Please note that biomarker implementation would employ probe-specific (gene-specific) setups, not whole-genome transcriptional quantitation).

### Model performance on the train and test set

The built models were tested on a randomly allocated sub datasets (0.7 train/0.3 test ratio). The data structure was partly imbalanced, i.e. the proportion of radiated samples versus controls. On the test set, most models achieved accuracy above 0.74, with only Logistic Regression and SIMCA scoring low with 0.56 and 0.42, respectively (**Table 2**). The highest accuracy was seen in the PDA, Elastic Net, PLS, and Naive Bayes (0.87 all), closely followed by KNN (0.86). Upon evaluation with the train set, the best model performance (no overfitting, stability) was achieved by KNN with an accuracy of 0.86, a kappa of 0.49, a sensitivity of 0.50, and a specificity of 0.94 using 5 predictors for dose and tissue classification. The second-tier models, i.e. Elastic Net, PLS, Naive Bayes, and Random Forest, all achieved the same accuracy with 0.86, but a decidedly lower kappa and sensitivity values of 0.35 and 0.25, respectively. (Please refer to **Table 2** for a comprehensive overview of performance values for all models.) Accordingly, the KNN model was integrated into the radiation biomarker identification pipeline.

### KNN-based biomarker performance and evaluation

The top 5 ranking biomarker candidates for dose prediction in kidney cortex and medulla with their relative importance levels (scaled between 0 and 100) are shown in **Table 5**. Tissue-specific biomarker candidates are given in **Table 6**. The identified top ranking biomarkers for cortex were *Cdkn1a (two probes)*, *Gria3*, *Mdm2* and *Plk2.* For medulla, *Brf2*, *Ccng1*, *Cdkn1a*, *Ddit4l*, and *Gria3* were top-ranking. Most of these genes were not identified in previous DEG analysis from the same data or proposed in radiation biomarker reviews. Among the seven identified genes (corresponding to 9 microarray probes with 1 probe identified in both tissues), *Brf2*, *Ddit4l*, and *Gria3* had a novelty score of 2 with no previous report in literature.

**Table 5:** Top-ranking biomarker candidates identified through the ML framework.

Previously Reported in								
Gene Symbol	ILMN ID	Prediction	Rank (Importance Level)	Conventional DEG Analysis*	Radiation Biomarker Reviews^§^	No. of References from Gene-Specific Literature Searches (String 1; String 2)	Main Biological Function	Novelty Score^†^
*Brf2*	ILMN_1258583	Dose Medulla	5 (0.0)	No	No	1; 2	Transcriptional initiation	2
*Ccng1*	ILMN_2702233	Dose Medulla	4 (13.6)	No	Yes	19; 33	Cell cycle regulation	0
*Cdkn1a*	ILMN_2634083	Dose Cortex	2 (91.3)	Yes	Yes	426; 1,151	Cell cycle regulation	0
		Dose Medulla	5 (100)					
	ILMN_2846775	Dose Cortex	1 (0.0)					
*Ddit4l*	ILMN_2695819	Dose Medulla	2 (44.8)	No	No	0; 2	Stress response (pro-apoptotic)	2
*Gria3*	ILMN_2638066	Dose Cortex	3 (19.2)	No	No	0; 1	Neurotransmission (glutamate receptor)	2
	ILMN_2638066	Dose Medulla	3 (19.4)					
*Mdm2*	ILMN_1250774	Dose Cortex	4 (5.2)	No	Yes	212; 926	Negative regulator of p53 (oncogene)	0
*Plk2*	ILMN_1260448	Dose Cortex	1 (100)	No	No	6; 10	Cell division (serine/threonine-protein kinase)	1

List of biomarker candidates (microarray probes) identified in the datasets (GSE44762) from murine kidney cortex and medulla irradiated with 0.13 – 13 Gy absorbed dose from i.v. injected ^177^Lu-octreotate over 24 hours including unirradiated controls. To determine the novelty of KNN-based biomarker candidates, the gene symbols were searched within previous conventional DEG analysis, within literature reviews on radiation biomarker candidates, and additional gene-specific literature searches in context with radiation and biodosimetry to determine whether the genes have been proposed for biodosimetry in previous work.

Relative importance levels scaled between 0 and 100.

No., number; ILMN, Illumina probe identifier; IR, ionizing radiation.

*As reported in Schüler et al., 2014 (22).

§ As reported in Snyder & Morgan (2004) (20), and Chaudhry (2008) (21).

^†^Novelty score: 0, reported as IR biomarker candidate in vast literature; 1, proposed as IR biomarker candidate in some literature; 2, not yet proposed as IR biomarker candidate in the literature.

**Table 6:** Top-ranking biomarker candidates for tissue-specificity identified through the ML framework.

Gene Symbol	ILMN ID	Prediction	Rank	Main Biological Function
*Atp2a3*	ILMN_2900462	Tissue	5	
*Itgav*	ILMN_1238119	Tissue	2	
*Napsa*	ILMN_2657867	Tissue	3	
*Pla1a*	ILMN_2722024	Tissue	1	
	ILMN_2974343	Tissue	4	

List of biomarker candidates (microarray probes) identified in the datasets (GSE44762) to predict tissue type. The dataset comprised murine kidney cortex and medulla irradiated with 0.13 – 13 Gy absorbed dose from i.v. injected ^177^Lu-octreotate over 24 hours including unirradiated controls.

ILMN, Illumina probe identifier.

**Figure 3:**
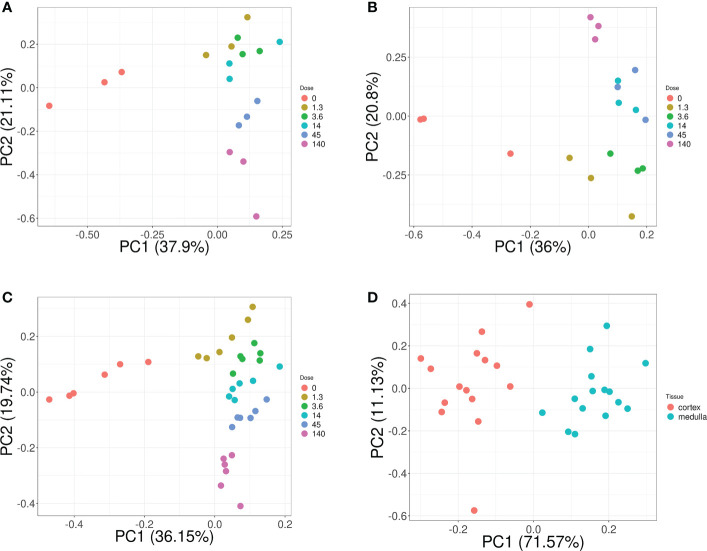
Dose and tissue specificity of KNN-based biomarker candidates in murine kidney tissues. Subsets of train and test data created from the transcriptomic microarray dataset GSE44762 (murine kidney cortex and medulla). The KNN method identified *Cdkn1a* (two probes), *Gria3*, *Mdm2* and *Plk2* for cortex, and *Brf2*, *Ccng1*, *Cdkn1a*, *Ddit4l*, and *Gria3* for medulla as radiation biomarker candidates between 0–13 Gy. (Please *cf.* **Table 5** for probe identifiers). The biomarkers successfully identified dose groups in kidney cortex **(A)**, kidney medulla **(B)**, and combined for both tissues **(C)**. *Atp2a3*, *Itgav*, *Napsa*, and *Pla1a* (two probes) were identified as tissue biomarker candidates achieved clear tissue separation across the PC1 dimension **(D)**. (Please *cf.* **Table 6** for probe identifiers.) Note that only radiation dose groups are included for tissue separation, i.e. 0 Gy control samples are excluded here.

**Figure 4:**
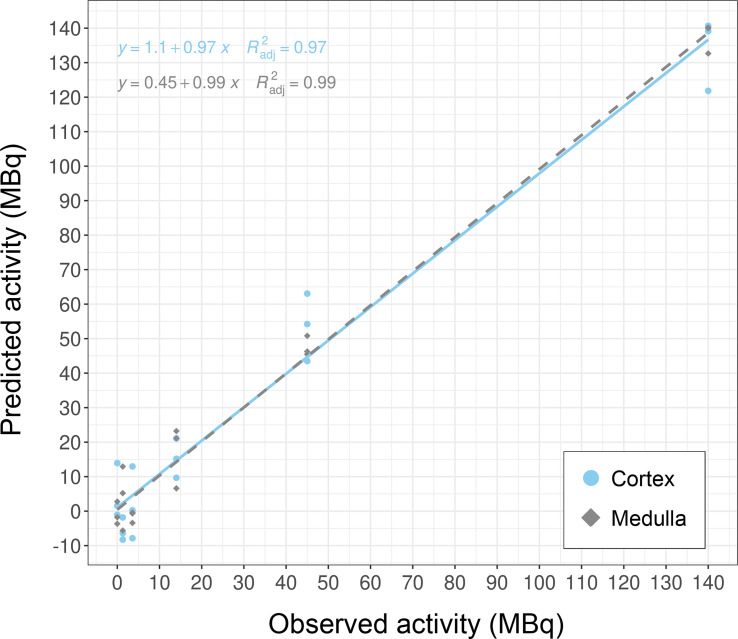
Dose prediction using KNN-based biomarker candidates in murine kidney tissues. The biomarker candidates (*Cdkn1a* (two probes), *Gria3*, *Mdm2* and *Plk2* for cortex; *Brf2*, *Ccng1*, *Cdkn1a*, *Ddit4l*, and *Gria3* for medulla (*cf.* **Table 5**)) predicted dose in kidney cortex and medulla in test data subsets (created from GSE44762). Note that units are given as injected activity to animals, i.e. 1.3–140 MBq ^177^Lu-octreotate, corresponding to 0.13–13 Gy to kidney tissue absorbed over 24 hours including unirradiated (0 Gy) controls.

Overall, these biomarker candidates achieved accurate categorization by dose and tissue as visualized by PCA analysis (**Figure 3**). There was a slight overlap in cortex tissue in the low dose range between 0.13 and 1.3 Gy, and in medulla tissue between 1.3 and 4.3 Gy. However, this overlap was resolved when cortex and medulla samples were analyzed together achieving clear separation. These candidates reproducibly differentiated the untreated (0 Gy) samples from any irradiated sample across PC1 by a relatively large distance. Similarly, the panel achieved clear tissue categorization and separation across PC1 irrespective of dose. The panel was also tested for dose prediction in each tissue via cross-validated multivariable dose regression analysis (**Figure 4**). Correlation between observed and predicted dose (injected activity) was high for both cortex and medulla with an R^2^ of 0.97 and 0.99, respectively. It should be noted, however, that the accuracy of dose prediction warrants further improvement, since error margins as observed for this data cohort are within the 1 Gy range, i.e. considered highly relevant for a radiobiological context (dose-response) and for biodosimetry (triage of unexposed vs. any exposed). Taken together, the biomarker panel performed well in feature categorization and dose prediction when tested in regression analysis.

## Discussion

ML includes a wide array of approaches and it is paramount to test a range of different methods in the exploratory phase to determine which one performs best on the data also considering resource demand for broad implementation. In our setting, GA/KKN performed well in terms of biomarker identification but drastically underperformed in terms of resource-efficiency. To avoid overfitting, feature selection and rigorous cross-validation was performed. We also closely monitored the performance measure sensitivity, specificity and kappa at each step. For possible feature clinical use model parsimony and simplicity was prioritized, wherefore feature selection was applied since the models showed similar performance after RFE was applied. In our study all models were built on the same uniformly preprocessed. Different models may perform better with different preprocessing methods, that is, model specific data preparation may lead to improved individual model performance.

The ML pipeline included training and testing stages that can be adapted to different features (experimental parameters) based on the given study design. While these steps can be processed automatically, data input still requires a manual step of phenotype file preparation from GSE (or similar) files. Since processor time was vastly reduced in the caret-based KNN approach, the algorithm can be easily trained for a new feature, e.g. radiation type or biological sex, or previously trained iterations can be readily tested on comparable datasets with the same features.

Our work further demonstrated the use of molecular biomarkers for dose prediction in normal tissues after radiation exposure. The identified candidate genes had highest sensitivity and specificity for the investigated irradiation setting, i.e. radiation exposure ranging from lower doses (0.13 and 0.34 Gy) over moderate doses (1.3, 4.3) to higher dose (13 Gy) protracted over 24 hours. Almost half of the genes in the dose biomarker panel are novel candidates with no previous radiation association in the literature. Brf2 (b-related factor 2) is the 50 kDa subunit of the RNA Polymerase III Transcription Initiation Factor and its regulation may represent the overall transcriptional orchestration of stress-induced (radiation-induced) cellular responses in the kidneys after 24 hours. Ddit4l (DNA-damage-inducible transcript 4-like) is a stress-responsive protein and a negative regulator of mTOR sensitizing towards cell death (25). Gria3 (glutamate receptor, ionotropic, AMPA3 (alpha 3)) is part of the glutamate receptor family that is predominantly involved in neurotransmission in the central nervous system. Recent work showed that Gria3 is also expressed in several peripheral tissues, including the kidneys, where the glutamate-receptor-transporter system appears to play a role in the pathogenesis of various diseases, which may be induced at higher radiation doses (26, 27). *Plk2* had a score of 1 with studies proposing its use in radiation biomarker panels for human lymphocytes after irradiation with gamma rays and alpha-particles emitted from astatine-211, as well as for primary human fibroblasts after gamma irradiation (28–30). Plk2 (polo like kinase 2) is a serine/threonine-protein kinase that functions in cell division and may indicate stress-induced regulation of proliferation. *Ccng1*, *Cdkn1a*, and *Mdm2* scored 0 as well-established radiation responsive genes with implementability in biomarker panels. Their main function resides within cell cycle regulation. Ccng1 (cyclin G1) regulates the activity of cyclin-dependent protein kinases (CDKs) for cell cycle regulation, while Cdkn1a (cyclin-dependent kinase inhibitor 1A) –also known as P21–functions as a regulator of cell cycle progression at G1 and S phase. Mdm2 (transformed mouse 3T3 cell double minute 2) is a key negative regulator of the tumor suppressor p53, which is involved in cell cycle regulation. Since Mdm2 functions as an oncogene, it can be speculated that its transcriptional gene regulation may indicate a very early step in the process of carcinogenesis initiated within 24 hours after irradiation. Data sets from long-term studies are needed to investigate whether these biomarker candidates are stable over time and can be assayed after days, weeks, or even months; or whether this candidate panel is time-point specific for 24 hours. In this context, it should be noted that radiation dose was protracted over time in contrast with acute dose delivery (single-dose over short time spans, i.e. seconds to minutes). This entails that a radiation dose-rate response underlies the gene expression response in the dataset, which may be specific for protracted dose. In the same light, the panel was identified for a radionuclide (lutetium-177) that emits high-energy beta radiation. The physical properties and resulting interactions with biomolecules differ between beta radiation and other types of ionizing radiation such as alpha-particles, X-rays, or accelerated heavy ions, which affects the biological response and in turn the biodosimetric efficacy of the candidate panel. In future work, it should be established whether the candidates function as a radiation- and time point-specific biomarker panel, or if they are general markers for other exposure and sampling settings.

Despite successful pipeline development, the ML approach still requires some refinement and model performance must be evaluated with similar datasets in future iterations. This quality assurance mostly concerns input data quality and experimental testing. The overlap in PCA clusters for certain dose groups is understood to result mainly from small sample size, i.e. statistical train/test issues, rather than underperformance of the chosen (KNN) ML method or identification process. Similarly, despite good correlation coefficients in regression, the dose accuracy for individual sample classification warrants further improvement to achieve high sensitivity and specificity in a biodosimetric setting where only a single sample (case study) is available that needs to be categorized accurately. While this error margin is a concern especially in the low dose regimen or between small dose differentials (here, 0 Gy vs. 0.13 Gy, and 0.13 Gy vs 0.34), it should be noted that PCA differentiated (clustered) unexposed samples from any irradiated sample across PC1 by comparably large distance. This is particularly important in the context of triage in a radiation hazard event: despite biological variation in gene expression levels in the absence of stressor (induction of pathway regulation), the untreated specimen clustered together reproducibly without overlap, i.e. for either tissue and when combined. Accordingly, if reliable reference samples could be established for biodosimetric purposes, a relative categorization could be achieved by a comparative approach.

Nevertheless, we conclude that further discovery optimization resides more in the experimental realm than in silico. It should be noted in this context that the input data was obtained from transcriptomic microarray assays where more than one nucleotide probe (sequence) can target the same mRNA for detection. In our setting, a radiation biomarker candidate (*Cdkn1a*) and a tissue biomarker (*Pla1a*) were identified by two variables, meaning different microarray probe sequences, which indicates splicing variants. RNA sequencing approaches have larger dynamic range than microarrays and are more accurate for quantitating splicing variants; despite the higher cost, sequencing data might elucidate whether these variants show differential performance at lower or higher dose intervals, or if they are fully redundant in their predictive performance. The strategic literature study revealed that most radiobiology study designs cover a certain radiation type, model system, and dose range, which limits the statistical power during training and subsequent testing, but also restricts the application of candidate panels on other exposure settings. Bearing in mind the budget cost for high-throughput study designs, the synergy field of radiation-bioinformatics would benefit from access to larger datasets with decidedly larger group sizes. Moreover, replicate studies are needed to validate (reproduce) biomarker candidates experimentally, ideally including setups where blinded test datasets are processed by the ML pipeline.

Moreover, the KNN method identified novel radiation-associated genes that have not been described in context with ionizing radiation before. Accordingly, the ML-pipeline may not only serve as an automated analytical platform, but also as an exploratory tool to unravel the complexity of radiation induced responses that have, for the most part, been investigated building on canonic pathways and *a priori* statements. Further, the pipeline should be trained (and tested) with fixed biological model parameters (species and strain of origin, specimen age and sex, etc.) but across different physical parameters such as radiation type, exposure settings, and dose intervals in order to elucidate the sensitivity and specificity of candidate panels in dependence of physical parameters. In essence, whether (certain) candidates are general biomarkers for any ionizing radiation exposure, or specific for a certain type of exposure. This question also translates to the time dimension; transcriptional biomarkers can have a comparatively short turnover and the stability or time-window of detection needs to be established for a given candidate panel. Conversely, time course-dependence can be employed to project time-of-exposure in hazard scenarios where time of irradiation is unknown but important for medical evaluation (triage) and decision-making.

With regard to the chosen omics data type, transcriptional gene expression has proven to be an effective molecular ecosystem for radiation biomarker discovery (11, 22, 31–43). From a bioinformatics perspective, training on sequencing data instead of microarray or on high-throughput protein mass-spectrometry (proteomics) data is comparable if phenotype files are properly curated and input data correctly formatted. Additional data type-specific pre-processing and filtering is required, but not a limitation for the ML framework as long as input datasets and variables are properly denoted. The ML pipeline could also be applied to samples (data) originating from non-normal tissues, for instance radiotherapeutic responses in irradiated tumors, but additional optimization during feature elimination and training is warranted. Cancer is a heterogeneous disease with large intra- and inter-tumoral variations, heterogeneous tumor microenvironments, and phenotype selection during the course of treatment, to name a few–all of which result in larger biological variation in gene expression. Accordingly, even larger cohorts are required compared with normal tissue datasets to achieve sufficient statistical performance. From a bioinformatics perspective, our framework can be easily adapted to other applications within the field of radiation research and radiotherapy.

## Conclusions

Caret-KNN drastically improved radiation biomarker identification and vastly reduced analytical cost. The established ML pipeline integrated gene expression data pre-processing with an optimized algorithm to automatically identify a gene panel for radiation dose prediction and tissue classification. The ML framework is a new technology in the field of radiation biodosimetry that can be translated to personalized radiotherapy and risk assessment. The next iteration will make the ML pipeline available online to lower cross-disciplinary barriers and facilitate broad implementation in the field of molecular biomarker discovery.

## Data availability statement

Publicly available datasets were analyzed in this study. This data can be found here: https://www.ncbi.nlm.nih.gov/geo/query/acc.cgi?acc=GSE44762. Caret can be retrieved from The Comprehensive R Archive Network at https://CRAN.R-project.org/package=caret. The R code for the ML framework is made available on GitHub at https://github.com/BrittaLangen/MLradiobiomarkers/blob/a8e13b50e354abe0ddd174243782170dc41eb33a/R%20code_ML%20pipeline%20radiobiomarkers.R.

## Author contributions

BA and BL share first authorship and are listed in alphabetic order. BL and ML conceived of the machine learning approach for radiation biomarker discovery and designed the study and framework development. BA, BL, and PL curated phenotype files. PL performed proof-of-concept with GA/KNN. BA performed framework development and implementation. ML and BL supervised machine learning approaches and implementation. BL performed literature searches and functional evaluation. All authors contributed to performance evaluation and data interpretation. BL drafted the manuscript. All authors contributed to writing and edited the manuscript.

## References

[1] Bates SE Longo DL. Tumor markers: value and limitations in the management of cancer patients. Cancer Treat Rev (1985) 12(3):163–207. doi: 10.1016/0305-7372(85)90037-4 2416441

[2] Sullivan JM Prasanna PG Grace MB Wathen LK Wallace RL Koerner JF. Assessment of biodosimetry methods for a mass-casualty radiological incident: medical response and management considerations. Health Phys (2013) 105(6):540–54. doi: 10.1097/HP.0b013e31829cf221 PMC3810609 24162058

[3] He K Zhang S Shao LL Yin JC Wu X Shao YW. Developing more sensitive genomic approaches to detect radioresponse in precision radiation oncology: from tissue DNA analysis to circulating tumor DNA. Cancer Lett (2020) 472:108–18. doi: 10.1016/j.canlet.2019.12.004 31837443

[4] Lacombe J Sima C Amundson SA Zenhausern F. Candidate gene biodosimetry markers of exposure to external ionizing radiation in human blood: a systematic review. PloS One (2018) 13:e0198851. doi: 10.1371/journal.pone.0198851 29879226 PMC5991767

[5] National Research Council. Health risks from exposure to low levels of ionizing radiation: BEIR VII phase 2. Washington, DC: The National Academies Press (2006). 25077203

[6] Preston RJ Boice JDJR Brill AB Chakraborty R Conolly R Hoffman FO. Uncertainties in estimating health risks associated with exposure to ionizing radiation. J Radiol Prot (2013) 33:573–88. doi: 10.1088/0952-4746/33/3/573 23803503

[7] Calabrese EJ. The threshold vs LNT showdown: dose rate findings exposed flaws in the LNT model part 1. The Russell-Muller debate. Environ Res (2017) 154:435–51. doi: 10.1016/j.envres.2016.12.006 28109526

[8] Mothersill C Seymour C. Implications for human and environmental health of low doses of ionising radiation. J Environ Radioact (2014) 133:5–9. doi: 10.1016/j.jenvrad.2013.04.002 23664231

[9] ICRP. Low-dose extrapolation of radiation-related cancer risk. ICRP Publ 99 Ann ICRP (2005) 35:(4). Available at: https://www.icrp.org/publication.asp?id=ICRP%20Publication%2099. 10.1016/j.icrp.2005.11.002 16782497

[10] Tubiana M. Dose-effect relationship and estimation of the carcinogenic effects of low doses of ionizing radiation: the joint report of the académie des sciences (Paris) and of the académie nationale de médicine. Int J Radiat Oncol Biol Phys (2005) 63:317–9. doi: 10.1016/j.ijrobp.2005.06.013 16168825

[11] Ghandhi SA Sinha A Markatou M Amundson SA. Time-series clustering of gene expression in irradiated and bystander fibroblasts: an application of FBPA clustering. BMC Genomics (2011) 12:2. doi: 10.1186/1471-2164-12-2 21205307 PMC3022823

[12] Sun R Limkin EJ Vakalopoulou M Dercle L Champiat S Han SR. A radiomics approach to assess tumour-infiltrating CD8 cells and response to anti-PD-1 or anti-PD-L1 immunotherapy: an imaging biomarker, retrospective multicohort study. Lancet Oncol (2018) 19(9):1180–91. doi: 10.1016/S1470-2045(18)30413-3 30120041

[13] Ardila D Kiraly AP Bharadwaj S Choi B Reicher JJ Peng L. End-to-end lung cancer screening with three-dimensional deep learning on low-dose chest computed tomography. Nat Med (2019) 25(6):954–61. doi: 10.1038/s41591-019-0447-x 31110349

[14] Mukherjee P Zhou M Lee E Schicht A Balagurunathan Y Napel S. A shallow convolutional neural network predicts prognosis of lung cancer patients in multi-institutional computed tomography image datasets. Nat Mach Intell (2020) 2:274–82. doi: 10.1038/s42256-020-0173-6 PMC8008967 33791593

[15] Yan L Zhan C Wang S Wang S Guo L. Genetic analysis of radiation-specific biomarkers in sinonasal squamous cell carcinomas. Tumour Biol (2016) 37(9):12001–9. doi: 10.1007/s13277-016-5057-3 27164935

[16] Karim S Mirza Z Chaudhary AG Abuzenadah AM Gari M Al-Qahtani MH. Assessment of radiation induced therapeutic effect and cytotoxicity in cancer patients based on transcriptomic profiling. Int J Mol Sci (2016) 17(2):250. doi: 10.3390/ijms17020250 26907258 PMC4783980

[17] Nia AM Khanipov K Barnette BL Ullrich RL Golovko G Emmett MR. Comparative RNA-seq transcriptome analyses reveal dynamic time-dependent effects of ^56^Fe, ^16^O, and ^28^Si irradiation on the induction of murine hepatocellular carcinoma. BMC Genomics (2020) 21(1):453. doi: 10.1186/s12864-020-06869-4 32611366 PMC7329445

[18] Li L Weinberg CR Darden TA Pedersen LG. Gene selection for sample classification based on gene expression data: study of sensitivity to choice of parameters of the GA/KNN method. Bioinformatics (2001) 17(12):1131–42. doi: 10.1093/bioinformatics/17.12.1131 11751221

[19] O'Connell GC Petrone AB Treadway MB Tennant CS Lucke-Wold N Chantler PD. Machine-learning approach identifies a pattern of gene expression in peripheral blood that can accurately detect ischaemic stroke. NPJ Genom Med (2016) 1:16038. doi: 10.1038/npjgenmed.2016.38 29263821 PMC5685316

[20] Snyder AR Morgan WF. Gene expression profiling after irradiation: clues to understanding acute and persistent responses? Cancer Metastasis Rev (2004) 23(3-4):259–68. doi: 10.1023/B:CANC.0000031765.17886.fa 15197327

[21] Chaudhry MA. Biomarkers for human radiation exposure. J BioMed Sci (2008) 15(5):557–63. doi: 10.1007/s11373-008-9253-z 18454354

[22] Schüler E Rudqvist N Parris TZ Langen B Helou K Forssell-Aronsson E. Transcriptional response of kidney tissue after ^177^Lu-octreotate administration in mice. Nucl Med Biol (2014) 41(3):238–47. doi: 10.1016/j.nucmedbio.2013.12.001 24434014

[23] R Core Team. R: a language and environment for statistical computing. Vienna, Austria: R Foundation for Statistical Computing (2021). Available at: https://www.R-project.org/.

[24] Max Kuhn M. Caret: classification and regression training. R package version 6 (2020) p. 0–86. Available at: https://CRAN.R-project.org/package=caret.

[25] Morquette B Morquette P Agostinone J Feinstein E McKinney RA Kolta A. REDD2-mediated inhibition of mTOR promotes dendrite retraction induced by axonal injury. Cell Death Differ (2015) 22(4):612–25. doi: 10.1038/cdd.2014.149 PMC4572858 25257176

[26] Dryer SE. Glutamate receptors in the kidney. Nephrol Dial Transplant (2015) 30(10):1630–8. doi: 10.1093/ndt/gfv028 25829324

[27] Du J Li XH Li YJ. Glutamate in peripheral organs: biology and pharmacology. Eur J Pharmacol (2016) 784:42–8. doi: 10.1016/j.ejphar.2016.05.009 27164423

[28] Turtoi A Schneeweiss FH. Effect of (211)At alpha-particle irradiation on expression of selected radiation responsive genes in human lymphocytes. Int J Radiat Biol (2009) 85(5):403–12. doi: 10.1080/09553000902838541 19382019

[29] Turtoi A Brown I Oskamp D Schneeweiss FH. Early gene expression in human lymphocytes after gamma-irradiation-a genetic pattern with potential for biodosimetry. Int J Radiat Biol (2008) 84(5):375–87. doi: 10.1080/09553000802029886 18464067

[30] Kis E Szatmári T Keszei M Farkas R Esik O Lumniczky K. Microarray analysis of radiation response genes in primary human fibroblasts. Int J Radiat Oncol Biol Phys (2006) 66(5):1506–14. doi: 10.1016/j.ijrobp.2006.08.004 17069989

[31] Amundson SA. Transcriptomics for radiation biodosimetry: progress and challenges. Int J Radiat Biol (2021) 21:1–9. doi: 10.1080/09553002.2021.1928784 PMC10026363 33970766

[32] Amundson SA. The transcriptomic revolution and radiation biology. Int J Radiat Biol (2022) 98(3):428–38. doi: 10.1080/09553002.2021.1987562 PMC9520858 34586968

[33] Larsson M Rudqvist N Spetz J Parris TZ Langen B Helou K. Long-term transcriptomic and proteomic effects in sprague dawley rat thyroid and plasma after internal low dose 131I exposure. PloS One (2020) 15(12):e0244098. doi: 10.1371/journal.pone.0244098 33382739 PMC7774980

[34] Langen B Rudqvist N Spetz J Helou K Forssell-Aronsson E. Deconvolution of expression microarray data reveals 131I-induced responses otherwise undetected in thyroid tissue. PloS One (2018) 13(7):e0197911. doi: 10.1371/journal.pone.0197911 30001320 PMC6042689

[35] Rudqvist N Spetz J Schüler E Parris TZ Langen B Helou K. Transcriptional response to 131I exposure of rat thyroid gland. PloS One (2017) 12(2):e0171797. doi: 10.1371/journal.pone.0171797 28222107 PMC5319760

[36] Langen B Rudqvist N Helou K Forssell-Aronsson E. Microarray studies on 211At administration in BALB/c nude mice indicate systemic effects on transcriptional regulation in non-thyroid tissues. J Nucl Med (2017) 58(2):346–53. doi: 10.2967/jnumed.116.176958 27765860

[37] Langen B Rudqvist N Spetz J Swanpalmer J Helou K Forssell-Aronsson E. Non-targeted transcriptomic effects upon thyroid irradiation: similarity between in-field and out-of-field responses varies with tissue type. Sci Rep (2016) 6:30738. doi: 10.1038/srep30738 27779251 PMC5078841

[38] Rudqvist N Spetz J Schüler E Langen B Parris TZ Helou K. Gene expression signature in mouse thyroid tissue after 131I and 211At exposure. EJNMMI Res (2015) 5(1):59. doi: 10.1186/s13550-015-0137-8 26492889 PMC4615992

[39] Rudqvist N Spetz J Schüler E Parris TZ Langen B Helou K. Transcriptional response in mouse thyroid tissue after 211At administration: effects of absorbed dose, initial dose-rate and time after administration. PloS One (2015) 10(7):e0131686. doi: 10.1371/journal.pone.0131686 26177204 PMC4503762

[40] Langen B Rudqvist N Parris TZ Schüler E Spetz J Helou K. Transcriptional response in normal mouse tissues after i.v. 211At administration - response related to absorbed dose, dose rate, and time. EJNMMI Res (2015) 5:1. doi: 10.1186/s13550-014-0078-7 25853007 PMC4384707

[41] Rudqvist N Schüler E Parris TZ Langen B Helou K Forssell-Aronsson E. Dose-specific transcriptional responses in thyroid tissue in mice after 131I administration. Nucl Med Biol (2015) 42(3):263–8. doi: 10.1016/j.nucmedbio.2014.11.006 25496975

[42] Schüler E Rudqvist N Parris TZ Langen B Spetz J Helou K. Time- and dose rate-related effects of internal 177Lu exposure on gene expression in mouse kidney tissue. Nucl Med Biol (2014) 41(10):825–32. doi: 10.1016/j.nucmedbio.2014.07.010 25156037

[43] Langen B Rudqvist N Parris TZ Schüler E Helou K Forssell-Aronsson E. Comparative analysis of transcriptional gene regulation indicates similar physiological response in mouse tissues at low absorbed doses from i.v. administered 211At. J Nucl Med (2013) 54:990–8. doi: 10.2967/jnumed.112.114462 23658216

